# Impact of Biologic Drugs on Comorbidity Outcomes in Rheumatoid Arthritis: A Systematic Review

**DOI:** 10.3390/jcm14134547

**Published:** 2025-06-26

**Authors:** Soumaya Boussaid, Rim Dhahri, Safa Rahmouni, Halil İbrahim Ceylan, Marwa Hassayoun, Maissa Abbes, Khaoula Zouaoui, Ismail Dergaa, Sonia Rekik, Nadia Boussaid, Imen Gharsallah, Raul Ioan Muntean, Hela Sahli

**Affiliations:** 1 Rheumatology Department, Rabta Hospital, Tunis 1007, Tunisia; soumaya.boussaid@fmt.utm.tn (S.B.); safarahmouni2015@gmail.com (S.R.); marwahass23@gmail.com (M.H.); maissa.abbes@fmt.utm.tn (M.A.); zouaoui.kha@gmail.com (K.Z.); rekik.sonia80@yahoo.fr (S.R.); sahlisrairihela@yahoo.fr (H.S.); 2 Faculty of Medicine of Tunis, University Tunis El Manar, Tunis 1007, Tunisia; rimdhahri@ymail.com (R.D.); imengharsallah2005@gmail.com (I.G.); 3 Research Unit LR 05 SP 01, La Rabta Hospital, Tunis 1007, Tunisia; 4 Rheumatology Department, Military Hospital of Tunis, Tunis 1006, Tunisia; 5 Department of Physical Education of Sports Teaching, Faculty of Sports Sciences, Atatürk University, 25240 Erzurum, Türkiye; 6 Higher Institute of Sports and Physical Education of Ksar Said, University of Manouba, Manouba 2010, Tunisia; phd.dergaa@gmail.com; 7 English Department, College of arts and humanities of Sousse, University of Sousse, Sousse 4002, Tunisia; nd.boussaid02@gmail.com; 8 Department of Physical Education and Sport, Faculty of Law and Social Sciences, University “1 Decembrie 1918” of Alba Iulia, 510009 Alba Iulia, Romania

**Keywords:** autoimmune management, biological therapy, chronic complications, comorbidity outcomes, hematologic impact, IL-6 antagonists, metabolic effects, TNF inhibitors

## Abstract

**Background:** The management of rheumatoid arthritis (RA) has advanced significantly with the introduction of biologic disease-modifying antirheumatic drugs (bDMARDs). Despite these therapeutic strides, RA prognosis remains profoundly affected by comorbid conditions, particularly cardiovascular and metabolic complications, which increase both morbidity and mortality. The role of bDMARDs in modulating comorbidities remains underexplored, with limited evidence on their effects across various non-RA conditions, such as respiratory, diabetic, and hematologic disorders. This systematic review aimed to evaluate the impact of bDMARDs on the progression and outcomes of comorbidities in RA patients, providing insights to guide personalized treatment approaches. **Methods:** This systematic review was registered in PROSPERO (CRD42022345903) and followed the PRISMA guidelines. Original research articles from PubMed and Scopus, published up to 18 July 2024, were included. Studies assessing the impact of bDMARDs on comorbidities in RA patients met the eligibility criteria. **Results:** A total of thirteen studies met the inclusion criteria. They were published from inception until July 2024. The studied comorbidities included pulmonary conditions (asthma, chronic obstructive pulmonary disease, and interstitial lung disease) (n = 2); diabetes (n = 3); anemia (n = 3); and malignancies (n = 3). The bDMARDs studied were tumor necrosis factor inhibitors (TNFis) (n = 9); Rituximab (n = 5); Tocilizumab (n = 5); Abatacept (n = 5); and Anakinra (n = 2). The most reported effects of bDMARDs on comorbidities were the following: (i) an exacerbation of pulmonary comorbidities for Abatacept and TNFis; (ii) patients switched to or initiated on Abatacept as their first targeted disease-modifying antirheumatic drug (tDMARD) showed directionally lower rates and costs of T2DM-related complications compared with patients switching to or initiating other tDMARDs; (iii) there was no difference between Abatacept and TNFis or Rituximab/Tocilizumab regarding diabetes treatment switching or intensification; (iv) Anakinra significantly reduced the HbA1c%; (v) decreased serum hepcidin levels and improvement in anemia were observed in patients treated with TNFis or Tocilizumab; and (vi) no decrease in overall survival time or the significant incident malignancy rate was noted in RA patients. **Conclusions:** Overall, bDMARDs appear safe for use in RA patients with comorbidities and may even provide specific benefits for conditions such as anemia and diabetes. These findings suggest that clinicians could consider tailoring biologic therapy based on each patient’s comorbidity profile, potentially enhancing both RA management and comorbidity outcomes. For instance, selecting biologics such as Anakinra or Tocilizumab might be advantageous for RA patients with concurrent diabetes or anemia, given their observed metabolic and hematologic benefits. This personalized approach could improve the quality of life and reduce healthcare costs by addressing RA and associated comorbidities more effectively.

## 1. Introduction

Rheumatoid arthritis (RA) is a chronic autoimmune condition characterized by chronic synovial inflammation and gradual joint destruction, which frequently causes disability and a lower quality of life [1,2]. Comorbidities in RA patients contribute to increased morbidity and mortality, leading to a reduced quality of life and a significant socioeconomic burden [3]. Biologic disease-modifying antirheumatic medicines (bDMARDs) have revolutionized RA treatment by targeting inflammatory cytokines including the following: (i) Tumor Necrosis Factor-alpha (TNF-α) inhibitors (TNFis), such as Adalimumab, Etanercept, Infliximab, Golimumab, and Certolizumab, block TNF-α, a pro-inflammatory cytokine central to RA pathogenesis, thereby reducing joint inflammation and damage [3,4]; (ii) Tocilizumab (TCZ), an interleukin-6 receptor (IL-6R) inhibitor, blocks IL-6 signaling—a key pathway implicated in the pathogenesis of rheumatoid arthritis—thereby reducing systemic inflammation [3,4]; (iii) Abatacept (ABA), a selective T-cell costimulation modulator, inhibits T-cell activation by binding to CD80/CD86 on antigen-presenting cells, disrupting the CD28-CD80/86 interaction [5]; (iv) Rituximab (RTX), a monoclonal antibody targeting CD20 on B cells, depletes B cells, which play a role in autoantibody production and inflammation [6]; and (v) Anakinra (ANA), an IL-1 receptor antagonist, inhibits IL-1β, a cytokine involved in RA and metabolic dysregulation [7,8].

In RA patients, bDMARDs have demonstrated efficacy in reducing disease progression, decreasing structural joint deterioration, and improving physical function [9]. However, many RA patients have comorbidities such as cardiovascular disease, respiratory disorders (such as interstitial lung disease), diabetes, anemia, and cancer, all of which complicate RA care and worsen the prognosis [6].

These comorbidities, whether resulting from systemic inflammation or the adverse effects of treatments, can alter the response to bDMARDs and impact overall patient outcomes [10]. On the other hand, the effects of bDMARDs on these coexisting diseases remain unclear.

Despite the therapeutic benefits of biologic agents in the management of RA, there are still conflicting results regarding their effect on pre-existing comorbidities. Some studies suggest that specific biologics, such as TNF inhibitors, may worsen pulmonary diseases in high-risk patients, mainly those with chronic obstructive pulmonary disease (COPD) and interstitial lung disease (ILD) [11]. Additionally, concerns exist about an increased risk of malignancy, as persistent immunosuppression may elevate the risk of certain malignancies [12,13]. On the other hand, the benefits of IL-6 inhibitors appear to extend to improved control of glucose and HbA1c levels in RA patients with type 2 diabetes mellitus (T2DM) [14]. The disparities in the effects of various bDMARDs on RA-associated comorbidities highlight the complexity of managing RA in patients with coexisting conditions. These findings suggest that the choice of bDMARDs should be tailored according to the patient’s comorbidity profile to optimize therapeutic outcomes.

The existing corpus of research lacks high-quality trials that accurately investigate the effects of bDMARDs on specific comorbidities in patients with RA. Most studies focus on RA-related outcomes, while comorbidities are often treated as secondary endpoints rather than primary objectives. Furthermore, observational studies frequently report the results of large populations without stratifying patients by comorbidity type or severity, making it difficult to understand how specific bDMARDs affect these conditions [15]. Differences in the study design, outcome measures, and definitions of comorbidities across studies hinder data synthesis and limit the reliability of evidence regarding the safety and efficacy of bDMARDs in patients with complex comorbidity profiles [16].

Given the limited availability of high-quality evidence, the scarcity of comorbidity-focused studies, and the inconsistencies among the existing findings, this systematic review aimed to evaluate the impact of bDMARDs on the progression and outcomes of specific comorbidities in RA patients, including respiratory, metabolic, hematologic, and malignancy-related conditions.

## 2. Methods

### 2.1. Protocol and Eligibility Criteria

This systematic review adhered to the guidelines set by the Preferred Reporting Items for Systematic Reviews and Meta-Analyses (PRISMA) [17]. The protocol was registered with the International Prospective Register of Systematic Reviews (PROSPERO) under the reference number (CRD42022345903).

The criteria for inclusion were developed using the following PICO tool questions [18]: P (population) = individuals with rheumatoid arthritis and at least one comorbidity; I (intervention/exposure) = exposure to bDMARDs; C (comparison) = absence of bDMARDs or comparisons among bDMARD molecules; and O (outcome) = outcomes related to comorbidities.

There were no limitations concerning the study design, setting, country, or timeframe. Our research utilized two databases: PubMed and Scopus, searched from their inception until 18 December 2024. All studies included involved patients diagnosed with rheumatoid arthritis (RA) who received bDMARD treatment and had at least one comorbidity. Only original articles in English were accepted. Publications that did not align with the objectives of this systematic review or did not constitute original research, such as reviews, editorials, qualitative studies, case reports, and letters to the editor, were excluded. Studies that focused on adverse events (e.g., infections and hypersensitivity reactions) as primary outcomes were also left out. In contrast, analyses concerning comorbidity-related outcomes, such as major adverse cardiovascular events (MACEs) in cardiovascular disease or glycemic control in diabetes, or other comorbidities (obesity, anemia, depression, osteoporosis, pulmonary conditions, or solid-organ cancers were applicable), were relevant, as these illustrate the progression of RA-related conditions rather than direct drug toxicity. Additionally, articles discussing the influence of bDMARDs on comorbidity risk factors and those focusing on infectious comorbidities were excluded.

### 2.2. Information Sources and Search

PubMed and Scopus were searched without any time constraints or filters. In PubMed, the search utilized keywords associated with “comorbidity” and “biologic drugs”. The keywords were selected based on the Medical Subject Headings (MeSH) terminology used in PubMed. The specific combination of Boolean operators and keywords can be found in Table 1. We compiled a list of comorbidities that are most often linked to RA, drawing on literature data [19,20]: diabetes (35.3%) [21], obesity (18 to 34%) [22], anemia (33 to 70%) [23], depression (17%) [24], cardiovascular disease (8.5%) [25], osteoporosis (6.3% to 36.3% for the hip) [26], pulmonary conditions (asthma (6.6%) and chronic obstructive pulmonary disease (3.6%)) [27], ulcers (1.2%) [28], and solid-organ cancers (5%) [19].

For PubMed, the search encompassed the article titles for all terms and the abstracts for those related to “comorbidity”. In Scopus, the search included article titles for all terms and abstracts and keywords for terms associated with the concept of “comorbidity”. Only studies involving humans were considered. The reference lists of selected articles were also reviewed. The first two authors of this systematic review came to a consensus on the articles to be included in this paper. All aspects of the systematic review methods were defined before commencing the review.

### 2.3. Study Selection

The first two authors of this systematic review independently gathered the data. They utilized a pilot-tested extraction form and resolved any disagreements through a consensus-based approach. During the initial online literature search, article titles were evaluated separately, followed by the elimination of duplicate articles. The abstracts of the selected articles were reviewed to see if they met the inclusion criteria. Full-text versions of the qualifying articles were then read to assess eligibility and retention. The extracted data encompassed the main methodological attributes of the articles, including study data (publication year, country, study design, number and average age of participants, inclusion and exclusion criteria, and evaluation methods), primary outcomes, and results. Any disputes between authors about eligibility were settled by consensus.

### 2.4. Methodological Quality Assessment

The assessment of methodological quality was conducted using the Joanna Briggs Institute (JBI) Critical Appraisal Tool, specifically the Checklist for Cohort Studies [29] and the Checklist for Randomized Controlled Trials (RCTs) [30].

The Checklist for Cohort Studies comprises 11 items focusing on (1) recruitment, (2) exposure measurement, (3) reliability of exposure measurement, (4) identification of confounding factors, (5) strategies to manage confounding factors, (6) ensuring participants were outcome-free at the study’s start, (7) validity and reliability of outcome measurement, (8) reported follow-up timeframe, (9) completeness of follow-up and strategies for handling incomplete follow-up, and (10) appropriate statistical analysis.

The RCT checklist consists of 13 items covering (1) randomization for participant assignment to treatment groups, (2) treatment group allocation, (3) baseline similarity of treatment groups, (4) participants’ blindness to treatment assignment, (5) blinding of those administering treatment to assignment, (6) blinding of outcome assessors to treatment assignment, (7) treatment group similarity apart from the intervention of interest, (8) completeness of follow-up and strategies for addressing incomplete follow-up, (9) ensuring participants were analyzed in their randomized groups, (10) consistent measurement of outcomes across treatment groups, (11) reliability of outcome measurements, (12) appropriate statistical analysis, and (13) appropriate trial design, including any deviations from standard RCT design. Each item on both checklists is scored as ‘yes’, ‘no’, ‘unclear’, or ‘not applicable’.

The first two authors independently evaluated the retained studies, resolving any discrepancies through discussion and reaching consensus on any disagreements regarding scoring.

## 3. Results

**Figure 1:**
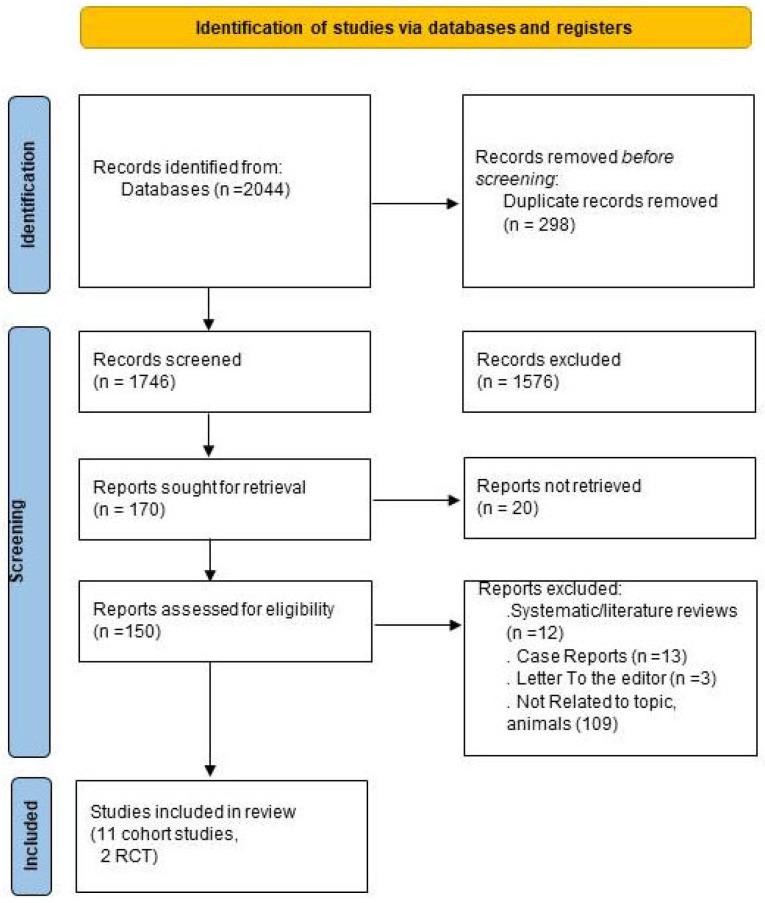
Flowchart outlining the protocol adopted in this systematic review.

The initial search resulted in 2044 papers. After removing duplicates, we screened 298 papers. During the title and abstract review, 1576 studies were excluded because they did not meet our objectives or were not published in English. From the remaining studies, 20 were removed for failing to meet the inclusion criteria. Additionally, 138 studies were excluded, including 12 systematic reviews, 13 case reports, 3 letters to the editor, and 110 studies unrelated to the topic and animals. Ultimately, 13 articles were retained [31,32,33,34,35,36,37,38,39,40,41,42,43]. Figure 1 presents the search results.

### 3.1. Methodological Quality Assessment Results

All thirteen studies [31,32,33,34,35,36,37,38,39,40,41,42,43] underwent evaluation for methodological quality. Regarding the cohort studies [31,32,33,34,35,37,38,40,41,42,43]: the number of items present in each study ranged from 7 [31,37,41,42] to 10 [32] out of a possible total of 11, with an average of 7.57. Each study included details on item 11. Only one study [42] addressed item 10, while the others lacked strategies for managing incomplete follow-up. All studies reported on items 2, 3, 4, 5, 7, 8, and 11. Five studies did not meet the criteria for item 1 [31,37,41,42,43]. Regarding the two RCTs [36,39], all items were recorded, except for items 4 and 5 [36,39] and item 6 in one study [39]. A summary of the methodology quality assessment for the cohort studies is presented in Table 2.

### 3.2. Study Selection and Characteristics

Nine studies compared groups of patients under bDMARDs [31,32,33,34,35,36,37,38,43]. Four studies compared RA under bDMARDs and RA without bDMARDs [39,40,41,42]. Among the studies included in our systematic review, two enrolled healthy control groups [38,39]. The studies were published between 2016 [41] and 2024 [32,33]. They were conducted in the United States [31,32,33,37,40,42], Italy [36], Japan [38,43], Egypt [39], Germany [34], and the UK [41]. Eight studies utilized national registry data [31,32,33,34,35,37,40,41]. The study design was reported in all studies [31,32,33,34,35,36,37,38,39,40,41,42,43]. Three designs were employed: a prospective design with repeated measures (n = 4) [31,33,34,38], a retrospective descriptive design (n = 7) [32,35,37,40,41,42,43], and a randomized controlled trial (RCT) (n = 2) [36,39]. Eleven studies reported the follow-up duration [31,32,33,34,35,37,39,40,41,42,43], which ranged from 1 [31] to 11 [37] years. All the studies mentioned the number of enrolled patients (39 to 153,788) [36,40] and the number of patients in the subgroup [31,32,33,34,35,36,37,38,39,40,41,42,43]. Eleven studies reported the patient’s age, which ranged from 42.04 ± 6.7 [34] to 73.7 ± 5.9 [35]. Respiratory comorbidities were reported in two studies [31,32], cardiovascular risk factors in two other studies [33,34], type 2 diabetes mellitus (T2DM) in three studies [35,36,37], anemia in three additional studies [37,38,39,40], and malignancy in three further studies [41,42,43]. The biologic drugs studied were TNFis [31,32,33,34,35,36,37,38,39,40,41,42,43], ABA [31,35,37,40,42,43], TCZ [32,33,35,37,38,40,42,43], ANA [35,36], and RTX [35,37,40,41,42]. In one study [42], bDMARDs other than TNFis were not specified. Table 3 exposes the main characteristics and methodological points of the retained studies.

## 4. bDMARD Impact on Comorbidities

### 4.1. bDMARD Impact on Respiratory Comorbidities

There were two studies [31,32] identifying RA patients with pulmonary comorbidities: interstitial lung disease (ILD), chronic obstructive pulmonary disease (COPD), and asthma. Patients were initiated on ABA or a TNF inhibitor (Adalimumab (ADA), Etanercept (ETA), Certolizumab Pegol (CTZ), Golimumab (GLM), or Infliximab (IFX)) [31,32]. Exacerbations requiring inpatient or emergency department visits occurred frequently after ABA or TNFi initiation, with no significant difference in the risk of ILD, COPD, or asthma exacerbation between the two treatments. RA-ILD patients treated with ABA may face a higher risk of mortality and mechanical ventilation compared to those receiving TNFis, especially among specific subgroups such as younger patients, those with cardiovascular risk factors, and ACPA-positive individuals [32].

### 4.2. bDMARD Impact on Cardiovascular Comorbidities

Two cohort studies investigated the effect of bDMARDs on cardiovascular comorbidities [33,34]. One examined the impact of bDMARDs on lipid changes, specifically LDL-C levels [33], while the other focused on the incidence of major adverse cardiovascular events (MACEs) [34].

- The first study concluded that TCZ increased LDL-C levels, and this increase was independent of clinical response. The Framingham score increased only with TCZ; however, risk scores remained stable across all biologics [33]. Moreover, despite the lipid increase associated with bDMARDs, there was no corresponding rise in cardiovascular risk, as indicated by the Reynolds Risk Score (RRS) [33]. The second study [29] found that JAK inhibitors (JAKis), bDMARDs, and conventional synthetic disease-modifying antirheumatic drugs (csDMARDs) did not increase the risk of major adverse cardiovascular events (MACEs) compared to TNFis [34].

### 4.3. bDMARD Impact on Diabetes

Three studies investigated the effect of bDMARDs on T2DM [35,36,37]: two cohort studies (n = 2) [35,37] and one RCT (n = 1) [36]. One study aimed to examine the impact of bDMARDs on T2DM-related consequences or complications [35]. Another study examined their effects on HbA1c%, and the remaining research examined their impact on treatment intensification [37]. The treatments assessed were TNFis (n = 3 studies) [35,36,37] and ANA (n = 2) [35,36]. RTX, TCZ, and ABA were investigated in two studies [35,37]. Sarilumab (a fully human anti-interleukin 6 (IL-6) receptor monoclonal IgG1 antibody) and Baricitinib (a Janus kinase (JAK) inhibitor) were examined in one study [35]. One study enrolled patients aged 65 years or older [35]. Patients switched to or initiating ABA as their first targeted disease-modifying antirheumatic drug (tDMARD) experienced directionally lower rates and costs of T2DM-related complications compared to patients switching to or initiating other tDMARDs [35]. ANA significantly reduced the HbA1c% level, while TNFi treatment did not [36]. There is no difference between ABA and TNFis, RTX, or TCZ regarding switching or the intensification of diabetes treatment [37].

### 4.4. bDMARD Impact on Anemia

Three studies dealt with anemia in RA patients [38,39,40]. Anemia was present at the index date in all patients in one study [39]. It was present in only 66% of patients in one study [38] and varied between 21 and 29% (according to the subgroups) in the remaining study [40]. Two studies examined the serum hepcidin-25 level and the hemoglobin (Hb) level after treatment with biologics [38,39], while one study considered only the Hb level [40]. Only one study specified that the authors investigated only anemia of chronic disease (ACD) [39]. The two other studies investigated anemia of different etiologies [38,40]. One study compared the impact of TCZ versus TNFi on anemia [38], another study compared the effect of ETN versus ADA [39], and the third study compared the effect of TCZ versus Tofacitinib or other bDMARDs [40]. Two studies enrolled a healthy control group [39]. All studies concluded that bDMARDs significantly increase Hb levels [38,39,40]. TNFis and TCZ improve anemia within 2 weeks [38]. The serum hepcidin level significantly decreases in patients treated with TNFis [5,6] and TCZ [38]. TCZ has shown the best results in improving anemia [38,40]. The decrease in serum hepcidin-25 levels in patients treated with TNFis was accompanied by a reduction in the serum IL-6 level [38].

A dramatic decrease in serum hepcidin-25 levels was observed after 4 months in patients treated with ETA or ADA, combined with methotrexate, and a significant improvement in anemia was noted (*p* < 0.05) [39].

### 4.5. bDMARD Impact on Solid Tumors

Three studies investigated the impact of bDMARDs on solid tumors [41,42,43]. They were cohort studies. One study examined the effect of bDMARDs on overall survival [42]. The two other studies examined the rate of incident malignancy (new primaries, local recurrences, and metastases) [41,43]. TNFis (without further specification) and RTX were investigated in two studies [41,42]. ETA, ADA, CTZ, GLM, ABA, and TCZ were studied in one study [42]. ABA was studied in one study [43]. Patients with RA and prior malignancy on TNFis or RTX did not have an increased risk of future incident malignancy [41]. ABA was as effective and safe in RA patients with and without a history of malignancy [43].

There was no significant difference in overall survival among RA patients treated with bDMARDs [42,43]. The use of TNFis was associated with improved survival, although the results were not statistically significant [42]. However, patients receiving non-TNFi agents had a 10% greater hazard compared with patients who did not receive any bDMARDs. The landmark analysis at 2—and 3-year time points after a cancer diagnosis shows numerically greater hazards in patients who received a bDMARD compared with patients who did not [42].

## 5. Discussion

This systematic review aimed to evaluate the effects of bDMARDs on specific comorbidities in patients with RA, including respiratory, metabolic, hematologic, and malignancy-related conditions. Our review indicated that bDMARDs were generally considered safe and effective for managing RA symptoms. Still, their impact on comorbidities required caution, as it varied based on the specific drug and the patient’s comorbidity profile.

We included 13 studies. According to the JBI critical appraisal tool, the methodological quality of the selected studies was considered “good,” with a mean score of 7.57 items. Although most studies have enrolled a large sample size of patients, the non-inclusion of a healthy control group in the majority of the studies [31,35,37,38,40,41,42,43] can be considered as a bias since the variations in the assessed parameters cannot be exclusively attributed to bDMARDs. In addition, due to the ‘real-life’ study design [31,35,37,38,40,41,42,43], the ongoing use of other medications, such as corticosteroids and methotrexate, could affect the outcome, as confounding factors were not exhaustively assessed (not all potential confounding factors were identified). Open design and unplanned interim analysis are more prone to bias than a double-blind controlled trial. Moreover, as most studies relied on diagnostic codes for patient assessment, there remains a potential risk of outcome misclassification. Another source of confusion is that treatments were prescribed based on clinical judgment in most cohort studies. Patients treated with one bDMARD were likely to differ from those treated with another bDMARD in multiple respects. As a result, comparisons between patients receiving different treatment regimens may be confounded by factors such as disease severity and baseline risk of the outcomes of interest. Unfortunately, meta-analyses were impossible due to the heterogeneous nature of the studies for each factor.

### 5.1. bDMARDs and Pulmonary Comorbidities

Regarding our review, only two studies assessed the impact of bDMARDs (TNFis and ABA) on pulmonary comorbidities [31,32]. The first one included RA patients with ILD, COPD, and asthma and found that pulmonary exacerbations were common among these patients, especially in the COPD group, regardless of the bDMARDs (ABA or TNFi) [31].

The other study focused on patients with RA-ILD and reported that ABA may be associated with a higher risk of mortality and mechanical ventilation compared to TNFis, particularly in specific subgroups such as younger patients, individuals with cardiovascular risk factors, and those who are ACPA-positive [32].

Biologic therapies in RA exhibit varying influences on pulmonary involvement, especially interstitial lung disease (ILD), a common and potentially serious extra-articular manifestation of the disease. TNFis have been associated with both new-onset and worsening ILD, although the evidence for a direct causal relationship remains inconclusive. In contrast, agents such as rituximab and ABA have shown a more favorable pulmonary safety profile, with reports indicating stabilization or even improvement in respiratory function. Tocilizumab presents mixed outcomes, with documented cases of both improvement and progression of ILD [44]. Some studies have reported increased mortality in patients with RA-ILD treated with TNF inhibitors (TNFis), raising concerns about the benefit–risk balance of this therapeutic class in such patients [45,46]. However, these studies have significant biases, warranting a reassessment of TNFi use in RA-ILD in light of ongoing scientific research.

Although less frequently reported than anti-TNF agents, Abatacept has been associated with both improvement and worsening of interstitial lung disease in patients with rheumatoid arthritis [47,48,49,50,51,52]. Case reports and small series suggest a relatively safer pulmonary profile, but exacerbations can still occur [53].

Rituximab has been the subject of encouraging therapeutic trials in interstitial lung diseases associated with other autoimmune conditions [54,55]. Additionally, four uncontrolled studies specifically involving RA-ILD patients (n = 133) demonstrated improvements in FVC and DLCO after the initiation of rituximab [56,57,58].

Some randomized controlled trials have been conducted to evaluate the efficacy of TNFis in treating COPD [59] or asthma [40], but most have not demonstrated any efficacy [59,60,61]. There may be confounding factors, particularly related to a history of smoking, the duration or severity of RA, and the severity of baseline lung conditions.

In clinical practice, the management of RA patients with pulmonary involvement requires an individualized approach to biologic therapy, guided by the nature and extent of interstitial lung disease, its progression, and associated comorbidities. Close collaboration between rheumatologists and pulmonologists plays a key role in developing effective treatment plans and reducing the risk of respiratory adverse effects. International societies such as the EULAR (European Alliance of Associations for Rheumatology) and the ACR (American College of Rheumatology) have not yet issued specific recommendations regarding the management of rheumatoid arthritis-associated interstitial lung disease (RA-ILD). However, the French Society of Rheumatology (Société Française de Rhumatologie, SFR) has provided clear guidance on this matter: “When a targeted synthetic DMARD (tDMARD) is needed in a patient with RA-ILD, abatacept or rituximab should be preferred, due to their more favorable pulmonary safety profile” [62].

### 5.2. bDMARDs and Cardiovascular Comorbidities

The effective control of disease activity by bDMARDs suggests a potential role of these agents in reducing cardiovascular risk [63]. However, the impact of bDMARDs on cardiovascular manifestations remains a topic of increasing interest. Several studies suggest that anti-TNF therapy may affect cardiovascular outcomes in patients with autoimmune diseases [64]. A recent review highlighted the dual nature of these effects: while anti-TNF agents generally offer a protective effect against cardiovascular events in this population, their use, particularly at high doses, in patients with pre-existing heart failure or a history of myocardial infarction may be ineffective or potentially harmful [65].

Additionally, clinical trials testing Etanercept in heart failure patients, such as RENAISSANCE and RECOVER, were prematurely terminated due to the lack of favorable effects on events, with a trend towards a deleterious impact in RENAISSANCE [66].

In this review, among the two enrolled studies, one examined the impact of bDMARDs on lipid changes, specifically LDL-C levels [33], while the other focused on the incidence of major adverse cardiovascular events (MACEs) [34].

In the first study, Tocilizumab was associated with increased LDL-C levels, regardless of the clinical response. However, the increase in lipid levels related to bDMARD treatment was not found to be linked to an increased CVD risk by the RRS. The latter remained stable across biologics. In contrast, Framingham’s score increased only with Tocilizumab [19]. It is well recognized that increases in serum total cholesterol, HDL-C, LDL-C, and triglyceride levels accompany tocilizumab administration. This has led to the recommendation of monitoring its use, particularly in patients with dyslipidemia and high CV risk.

The second study evaluated four treatments: JAKis (baricitinib, filgotinib, tofacitinib, or upadacitinib), TNFis (adalimumab, certolizumab, etanercept, golimumab, or infliximab), other bDMARDs (abatacept, rituximab, sarilumab, or tocilizumab), and csDMARDs [34]. This study provided real-world evidence that the incidence of MACEs in patients with RA was similar across all groups [34]. However, adverse cardiovascular events were not stratified in the other bDMARD group. Thus, the impact of Tocilizumab was not evaluated separately.

In summary, while anti-TNF therapy has shown benefits in treating inflammatory diseases, its effects on cardiovascular health are complex and may vary depending on the patient’s underlying cardiovascular condition. Tocilizumab should be avoided in patients with a high CV risk.

### 5.3. bDMARDs and Diabetes

We included three studies evaluating the impact of bDMARDs on diabetes [35,36,37], all of which reported a beneficial effect.

Patients receiving biologic therapies (ABA, TNFi, RTX, TCZ) did not require modification or intensification of their antidiabetic treatment, which may suggest that these therapies do not adversely affect glycemic control [37]. On the contrary, bDMARDs such as Anakinra significantly reduced HbA1c levels [36]. ABA was associated with lower rates and costs of T2DM-related complications than other TDMARDs [35].

Biologic DMARDs have been reported to have beneficial effects on glucose metabolism, including reductions in glucose and insulin levels, as well as significant improvements in the HbA1c percentage [67]. Other authors have reported a beneficial effect of an IL-1 inhibitor (in RA patients with T2D) on the HbA1c rate, confirming that Anakinra could improve insulin secretion [68,69]. Furthermore, clinical studies have demonstrated that IL-1 inhibitors and TNF inhibitors improve glucose metabolism through their anti-inflammatory actions [68]. A recent hypothesis suggests that ABA treatment may directly influence glucose metabolism by increasing insulin sensitivity [67]. Indeed, RA patients treated with ABA, compared to those treated with TNFi, had a lower risk of incident diabetes mellitus [67]. Evidence suggests that inhibiting T cell costimulation reduces the effect of T cells in adipose tissues and improves regulatory T cell function, which is thought to improve insulin sensitivity [70]. Moreover, observational reports have shown improvements in HbA1c% in RA patients treated with TCZ [71,72], supporting the preclinical evidence of IL-6’s key role in inflammation and metabolism [73]. Thus, ANA and ABA should be preferred in RA patients with T2DM.

### 5.4. bDMARDs and Anemia

While both TNFis and TCZ were associated with decreased serum hepcidin-25 levels and a significant improvement in anemia, the effects were more pronounced with TCZ [39]. The decrease in serum hepcidin-25 levels in patients treated with TNFis is accompanied by a reduction in the serum IL-6 level [38]. These findings suggest that IL-6 suppression may contribute to the improvement in anemia.

The findings of this review align with those of Doyle et al. [73]. Indeed, the authors, through a pooled analysis of three large, multicenter, double-blind, randomized clinical trials, concluded that treatment with IFX plus MTX significantly improved hemoglobin levels among anemic RA patients compared to treatment with placebo plus MTX, regardless of disease activity improvement [74].

According to the studies, the prevalence of anemia in RA patients ranges from 30 to 70% [75,76]. CDA and iron deficiency anemia are considered the most common [77]. Treatments with anti-cytokine agents such as IFX, TCZ, and ANA have been shown to significantly decrease serum Hb levels in RA patients [78,79,80,81,82]. Indeed, TNF-α, IL-6, and IL-1 are thought to contribute to RA-anemia development by modulating iron metabolism and suppressing bone marrow erythropoiesis [82,83,84], particularly by affecting the serum hepcidin level.

In a recent meta-analysis, the authors demonstrated that RA patients with anemia have higher serum hepcidin levels than those without anemia (SMD = 0.400, 95% CI = 0.080 to 0.720, *p* = 0.014) [85]. Indeed, hepcidin, a type II acute protein, is an inflammatory mediator produced in response to inflammatory cytokines such as interleukin-6 [86,87]. Its expression is regulated by inflammation, anemia, and hypoxia [88]. In addition to its inflammatory action, it modulates the iron-regulating hormone by binding to the ferroportin iron transporter and inducing its internalization and degradation [89,90].

In summary, TNFis and TCZ should be considered as therapeutic options for RA patients with anemia, with tocilizumab being the preferred agent due to its superior efficacy.

### 5.5. bDMARD and Solid Tumors

Although the efficacy of bDMARDs has been firmly established, concerns remain regarding their long-term safety, specifically the potential risk of malignancy [91]. Indeed, an increased risk of malignancies, particularly lymphoma, has been reported in some studies [91]. However, this increased risk may be attributable to the underlying chronic disease, the severity of the condition, and the use of concomitant medications.

The putative association between bDMARDs and malignancy is hard to establish given the complex role of cytokines, lymphocytes, and other elements of immune competence in cancer pathogenesis. For instance, TNF-α has both anti- and pro-cancer effects [92]. Similarly, IL-6 directly promotes cancer cell proliferation by activating signal transducer and activator of transcription 3 (STAT3), which drives cell cycle progression [93].

In this review, all three included studies concluded that bDMARDs are safe [40,41,42]. Patients with RA and prior malignancy on TNFis, RTX, and ABA did not have an increased risk of future incident malignancy [40,42]. ABA was as effective and safe in RA patients with a history of malignancy as in those without [42].

Nevertheless, these results must be interpreted with caution. The median follow-up duration was 6.8 years [40]. Thus, the rate of IM could be increased with a longer follow-up duration. Furthermore, the small number of each type of IM does not allow for the analysis of specific cancer risks. Another limitation is that various potential confounding factors could be a source of bias: patients’ baseline characteristics, the disease activity (which may be responsible for an underlying inflammation, leading to malignant transformation), the primitive cancer type and extension, and factors associated with prior cancer that led to a decision not to start a biologic in certain patients [40].

Regarding ABA, an international, up to 10-year observational safety study assessed the incidence of malignancy in four real-world data sources: two bDMARD registries (the Anti-Rheumatic Therapy in Sweden register and the Rheumatoid Arthritis Observation of Biologic Therapy German registry), a disease registry (The National Databank for Rheumatic Diseases in the USA), and an administrative health claims database (the population-based British Columbia Canadian RA Cohort [94].

The authors demonstrated that ABA was not associated with a significantly increased overall cancer risk compared to cs/b/tsDMARDs, (breast cancer or lung cancer). However, the Swedish registry showed a significantly increased risk of lymphoma with ABA vs. b/tsDMARDs. Recently, the EULAR Task Force formulated five overarching principles and eight points to consider before the initiation of targeted therapies in patients with inflammatory arthritis and a history of cancer [95]. In patients with cancer in remission, targeted therapy should not be delayed. However, if the tumor is not in remission and the RA is active, a shared decision-making process is necessary between the patient, rheumatologist, and oncologist. The EULAR TASK Force recommended targeted therapy, depending on the clinical context. B-cell-depleting therapy may be preferred in patients with a history of lymphoma, while TNFis may be preferred in patients with a history of solid cancer. JAKis and ABA should only be considered in the absence of other therapeutic alternatives, due to the lack of data regarding their safety in patients with a history of malignancy.

### 5.6. bDMARD Impact on Osteoporosis and Depression

RA is often complicated by comorbidities such as osteoporosis and depression, which impair quality of life, complicate management, and worsen functional prognosis [96,97]. The direct impact of biologics on these comorbidities remains poorly documented. We conducted a targeted review based on the PICO criteria, which did not identify any articles that strictly met these criteria. However, several publications provide indirect, valuable evidence for understanding these comorbidities in RA.

a. Osteoporosis

Osteoporosis is a frequent comorbidity in RA, driven by chronic systemic inflammation, reduced mobility, and long-term glucocorticoid use. In a systematic review and meta-analysis using Mendelian randomization, Ji et al. [96] investigated genetic determinants of bone mineral density and fracture risk. Although not RA-specific, this study highlights the multifactorial nature of osteoporosis, suggesting that therapeutic control of inflammation alone, such as with biologics, may not be sufficient to mitigate bone loss fully. These findings reinforce the importance of incorporating targeted osteoporosis prevention strategies into the overall care of RA patients.

b. Depression

Biologic therapies, particularly TNFis, have been extensively studied for their impact on depression in patients with RA. Evidence suggests that TNFis may reduce the risk of depression by controlling inflammation, which is known to contribute to depressive symptoms in RA [97]. However, some reports indicate that the initiation or switching of biologic therapies can be associated with increased psychological distress and the use of antidepressants, reflecting a complex and variable effect on mental health [98]. Specifically, etanercept has been shown to have a more favorable profile regarding depression risk compared to infliximab [99]. Additionally, biologics improve quality of life and psychological well-being in RA patients [100]. Social support further enhances these outcomes, highlighting the importance of integrating psychosocial care into RA management [101]. Despite these findings, our PICO-based literature search found no studies directly addressing the impact of biologic therapies on osteoporosis and depression outcomes in RA, revealing a significant gap for future research.

### 5.7. Potential Role of Genetic and Ethnic Variability in Treatment Response and Safety Profile

The response to biologic therapies, such as TNF inhibitors, rituximab, and tocilizumab, varies significantly according to patients’ genetic backgrounds. Polymorphisms in Fc gamma receptors, notably FCGR3A F158V and FCGR2A R131H, influence treatment efficacy by modulating systemic inflammation, a key factor in reducing cardiovascular and metabolic risks [102,103]. Genotype-guided therapy holds promise for optimizing both articular and systemic outcomes. In parallel, epigenetic modifications contribute to the persistence of chronic inflammation by activating synovial fibroblasts and immune cells, thereby accelerating the progression of comorbidities. Biologic agents that target these pathways may indirectly help mitigate complications such as atherosclerosis and osteoporosis [104]. However, no single biomarker has yet proven reliable in predicting the therapeutic response to anti-TNF agents or their impact on associated comorbidities. This underscores the importance of multi-marker strategies for truly personalized treatment approaches [105]. Moreover, ethnicity-related genetic variations influence both the efficacy and safety of biologics. Specific allelic variants—more prevalent in Asian or Caucasian populations—can partly explain the interethnic differences in treatment response and adverse effects, including susceptibility to infections. Therefore, integrating genetic and ethnic factors into clinical decision-making is crucial for optimizing therapeutic outcomes and reducing risk [102,103].

Integrating genetic, epigenetic, and clinical data can enhance personalized medicine approaches, improving the control of both RA symptoms and associated comorbidities, thereby enhancing patient quality of life [106].

To the author’s knowledge, no previous systematic review has focused on the effect of bDMARDs on RA comorbidities. We demonstrated that bDMARDs may enable the therapeutic targeting of both disorders (RA and some comorbidities), and using a single agent may help manage both inflammatory and metabolic diseases. However, this result should be interpreted cautiously, given that only thirteen studies were included in this systematic review.

## 6. Limitations

This review has several limitations. We explicitly acknowledge a key limitation of our review: it was necessarily constrained by the current literature’s availability of evidence. While our selection of comorbidities was systematic and guided by EULAR recommendations and existing data, many other potential comorbidities were not evaluated due to a lack of available studies. This absence reflects a gap in the literature rather than a limitation of our search strategy. Moreover, most included studies were cohort studies, introducing potential selection bias in prescribing bDMARDs. In addition, there were considerable differences in sample sizes and the proportions of bDMARD users across studies; notably, three studies [34,36,38] had small sample sizes, which limited their ability to detect significant associations. Third, most studies compared biological drugs without a control group, which may have introduced bias in the findings. Additionally, this review only covered four types of comorbidities, as studies meeting our inclusion criteria were not available for other conditions. While these findings offer valuable insights, they should be confirmed by rigorous RCTs. However, such trials are challenging to conduct due to ethical and funding constraints and the need for extended follow-up periods.

## 7. Conclusions

Our systematic review highlights the complexities of managing rheumatoid arthritis in patients with multiple comorbidities, underscoring the importance of a personalized therapeutic approach tailored to individual needs. Identifying and consistently monitoring comorbidities before and during RA treatment is critical to improving patient outcomes. This study suggests that bDMARDs can be carefully chosen depending on individual comorbidities, potentially providing dual advantages in addressing both RA and related illnesses such as anemia and diabetes. Our findings support a tailored approach that takes into account both the inflammatory and metabolic elements of RA, as well as its comorbidities:

- ABA may stabilize or even improve lung function. It should not be systematically avoided in patients with interstitial lung disease associated with RA (ILD-RA). It remains a valid treatment option alongside TNF inhibitors, anti-CD20, and IL-6 inhibitors, depending on the patient’s clinical profile.
- ABA and, to a lesser extent, anakinra, may have beneficial effects on glucose metabolism and could help reduce the risk of developing type 2 diabetes. However, these findings need further confirmation through large-scale controlled studies.
- Despite its impact on lipid profiles, TCZ has not been consistently associated with increased cardiovascular events. It may be considered in patients with high cardiovascular risk, provided close monitoring is implemented.
- TNFis and TCZ should be considered in patients with anemia, with TCZ being the preferred agent due to its superior efficacy.
- Targeted therapies should not be delayed in patients with cancer in remission. In active RA with ongoing malignancy, treatment should follow shared decision-making. In patients with a history of malignancy in remission, anti-CD20 is recommended for prior lymphoma and TNFis for solid tumors, while JAK inhibitors and abatacept should only be considered for cases lacking safer alternatives.

More rigorous RCTs are needed to address the existing gaps and enhance therapeutic decision-making. These studies should focus on comorbidity-related outcomes, employ standardized measures, and provide robust evidence to refine treatment guidelines, particularly for patients with complex comorbidity profiles. Additionally, further research is needed to better elucidate the effects of biologic therapies on a broader range of comorbidities.

## Figures and Tables

**Table 1:** Applied terms in the present systematic review.

Terms Related to Comorbidities	Terms Related to Biologic Drugs
(comorbidities [Title/Abstract] OR comorbidity [Title/Abstract] OR cardiovascular [Title/Abstract] OR osteoporosis [Title/Abstract] OR pulmonary [Title/Abstract] OR depression [Title/Abstract] OR ulcer [Title/Abstract] OR diabetes [Title/Abstract] OR obesity [Title/Abstract] OR anemia [Title/Abstract] OR Malignancies [Title/Abstract] OR malignancy [Title/Abstract] OR tumor [Title/Abstract])	AND (biologic [Title] OR TNF [Title] OR certolizumab [Title] OR infliximab [Title] OR etanercept [Title] OR adalimumab [Title] OR golimumab [Title] OR rituximab [Title] OR tociluzimab [Title] OR anakinra [Title] OR IL1 [Title] OR abatacept [Title] OR CTLA4 [Title]) AND (Rheumatoid arthritis [Title] OR (RA [Title])

**Table 2:** Methodology quality evaluation of the cohort studies by the Joanna Briggs Institute Critical Appraisal Tool.

	1	2	3	4	5	6	7	8	9	10	11
Risk of exacerbation of pulmonary comorbidities in patients with rheumatoid arthritis after initiation of abatacept versus TNF inhibitors: A cohort study [31]	N	Y	Y	Y	Y	N	Y	Y	U	N	Y
Abatacept versus tumor necrosis factor inhibitors on mortality and medical utilizations in the treatment of rheumatoid arthritis associated interstitial lung disease: a large-scale real-world retrospective cohort study [32]	Y	Y	Y	Y	Y	Y	Y	Y	Y	N	Y
Effect of biologic agents and inflammation on lipid levels and cardiovascular risk in rheumatoid arthritis patients [33]	Y	Y	Y	Y	Y	N	Y	Y	Y	N	Y
Risk of major adverse cardiovascular events in patients with rheumatoid arthritis treated with conventional synthetic, biologic and targeted synthetic disease-modifying antirheumatic drugs: observational data from the German RABBIT register [34]	Y	Y	Y	Y	Y	N	Y	Y	U	N	Y
Diabetes-Related Complications and Costs in Medicare Beneficiaries with Comorbid Rheumatoid Arthritis and Diabetes Treated with Abatacept Versus Other Targeted DMARDs [35]	Y	Y	Y	Y	Y	N	Y	Y	U	N	Y
Anti-interleukin-1 treatment in patients with rheumatoid arthritis and type 2 diabetes [TRACK]: A multicentre, open-label, randomised controlled trial [36]	Y	Y	Y	N	N	Y	Y	Y	Y	Y	Y
Use of biologic or targeted-synthetic disease-modifying anti-rheumatic drugs and risk of diabetes treatment intensification in patients with rheumatoid arthritis and diabetes mellitus [37]	Y	Y	Y	Y	Y	N	Y	Y	Y	N	Y
Comparative evaluation of the effects of treatment with tocilizumab and TNF-α inhibitors on serum hepcidin, anemia response and disease activity in rheumatoid arthritis patients [38]	Y	Y	Y	Y	Y	N	Y	Y	Y	N	Y
The effectiveness of etanercept and adalimumab on anemia of chronic disease and serum hepcidin in patients with rheumatoid arthritis, a comparative study [39]	Y	Y	Y	N	N	N	Y	Y	Y	Y	Y
Effectiveness of Biologic and Non-biologic Antirheumatic Drugs on Anaemia Markers in 153,788 Patients with Rheumatoid Arthritis: New Evidence from Real-World Data [40]	N	Y	Y	Y	Y	N	Y	Y	N	N	Y
The incidence of cancer in patients with rheumatoid arthritis and a prior malignancy who receive TNF inhibitors or rituximab: results from the British Society for Rheumatology Biologics Register. Rheumatoid Arthritis [41]	N	Y	Y	Y	Y	N	Y	Y	N	N	Y
Overall survival in patients with rheumatoid arthritis and solid malignancies receiving biologic disease-modifying antirheumatic therapy [42]	N	Y	Y	Y	Y	N	Y	Y	N	Y	Y
Efficacy and safety of abatacept in patients with rheumatoid arthritis with previous malignancy [43]	N	Y	Y	Y	Y	Y	Y	Y	N	N	Y

N: No; U: Unclear; Y: Yes; Item 1: Were the two groups similar and recruited from the same population?; Item 2: Were the exposures measured similarly to assign people to both exposed and unexposed groups?; Item 3: Was the exposure measured in a valid and reliable way?; Item 4: Were confounding factors identified?; Item 5: Were strategies to deal with confounding factors stated?; Item 6: Were the groups/participants free of the outcome at the start of the study (or at the moment of exposure)?; Item 7: Were the outcomes measured in a valid and reliable way?; Item 8: Was the follow-up time reported and sufficient to be long enough for outcomes to occur?; Item 9: Was follow-up complete, and if not, were the reasons for loss to follow-up described and explored?; Item 10: Were strategies to address incomplete follow-up utilized?; Item 11: Was appropriate statistical analysis used?

**Table 3:** The main characteristics and results of the selected studies aiming to evaluate the effects of biologic drugs on comorbidity outcomes in RA.

Author/ Year of Publication/ Country [Ref]	Study Design/ Population/(Age (Year), n)	Register	Selection Criteria/Comorbidities	Main Outcomes/Follow-Up Duration	Results
**Kang E.H.** **2020** **US** [34]	Prospective cohort study. ILD cohort: ABA (n = 420)/TNFi (n = 1579). COPD cohort: ABA (n = 862)/ TNFi (n = 3693). Asthma cohort: ABA (n = 478)/ TNFi (n = 2108).	U.S. Medicare +Truven MarketScan databases	***Inclusion criteria***: RA patients with ILD, COPD, or asthma who initiated ABA or a treatment in the index period. ***Exclusion criteria***: Patients who used anakinra, RTX, TCZ, or Tofa in the pre-index period/ID. Nursing home residents. Patients with malignancy, cystic fibrosis, HIV infection, radiation oncology services, or renal dialysis.	**Main outcomes**: Severe exacerbations requiring hospitalization, ED, or outpatient visits due to a specific pulmonary condition. Follow-up time started on the day after the I-D and continued through to the earliest date of discontinuation. **Follow-up duration**: (1 to 1.6 years).	While pulmonary exacerbations were common, especially in COPD, **ABA and TNFis showed similar risks** for worsening lung disease. Hospitalizations/ED visits were frequent, emphasizing the need for close monitoring.
**Shih P.-C.** **2024** **US** [32]	Retrospective cohort study. 34,388 RA-ILD patients were identified; 895 patients matched in each group (ABA and TNFi) after propensity score matching.	The TriNetX electronic health record database	***Inclusion criteria***: Diagnosis of RA-ILD, new prescription for ABA or TNFi. Comorbidities: cardiovascular risk factors. ***Exclusion criteria***: not mentioned.	**Primary outcome**: All-cause mortality.	**Mortality**: The ABA group exhibited a higher risk of all-cause mortality (HR 1.296; 95% CI 1.006–1.671). **Mechanical Ventilation**: Increased risk in ABA-treated patients aged 18–64 years (HR 1.853; 95% CI 1.002–3.426) and those with cardiovascular risk factors (HR 2.015; 95% CI 1.118–3.630).
**Pappas D.** **2024** **US** [33]	Prospective comparative effectiveness study with 1698 patients.	CERTAIN (CorEvitas RA registry (US-based).	***Inclusion criteria***: RA with moderate to high disease activity, completed 6-month follow-up, had available lipid levels at baseline and follow-up, and were initiating bDMARD therapy. ***Exclusion criteria***: Missing lipid data or follow-up visits (1097 excluded). Selection: Diagnosis of RA, initiating bDMARD therapy, moderate to high disease activity at baseline.	**Main outcomes**: Lipid changes (LDL-C levels). Cardiovascular risk (Reynolds Risk Score [RRS], Framingham risk score). Relationship between CRP changes and lipid changes. **Follow-up duration**: Baseline, 3 months, and 6 months post-initiation. Comorbidities: Focus on cardiovascular risk and systemic inflammation (CRP, LDL-C levels).	Lipid changes: Tocilizumab increased LDL-C; increases were independent of clinical response. Risk scores: RRS stable across biologics. Framingham’s score increased with tocilizumab. Lipid increases on bDMARDs do not increase cardiovascular risk by RRS.
**Meissner Y.** **2023** **Germany** [34]	Observational cohort study. Total Participants: 14,203. Treatment episodes (21,218 patient-years). Analysis period: Episodes of DMARD treatment initiated between January 2017 and April 2022.	German RABBIT register	***Inclusion criteria***: Patients with established RA who initiate a new DMARD treatment. ***Exclusion criteria***: Not mentioned.	**Main outcome**: Incidence of major adverse cardiovascular events (MACEs). **Follow-up duration**: Not specified.	JAKis: No increased risk of MACEs compared to TNFis. Biologic bDMARDs: No increased risk of MACEs compared to TNFis. csDMARDs: No increased risk of MACEs compared to TNFis.
**Patel V.** **2022** **US** [35]	Retrospective cohort study (2009–2017) 8105 RA + T2DM. TNFi-experienced group: 2169 ABA/TNFi and 2118 ABA/other non-TNFi. tDMARD-naïve group: 2667 ABA/TNFi and 2247 ABA/other non-TNFi.	Medicare FFS	***Inclusion criteria***: RA≥ 65 years. T2DM or use of antidiabetic drugs before initiating tDMARD. TNFi-experienced patients: required to have used a prior (but not the same) TNFi in the 12-month pre-index period and switched to a subsequent different tDMARD treatment. tDMARD-naïve patients: initiated either ABA, TNFi, or other non-TNFi as their first tDMARD. ***Exclusion criteria***: Patients with evidence of type 1 diabetes or cancer during a 12-month pre-index period. Patients treated with more than one tDMARD on the index or had prior dispensing for the index drug within 1 year before ID.	**Main outcomes**: T2DM-related HCRU and costs in the follow-up period T2DM-related complications (retinopathy, nephropathy, neuropathy, cerebrovascular, cardiovascular, peripheral vascular disease, and glucose complications). **Follow-up duration**: Until the occurrence of one of the above events	Patients treated with ABA (abatacept) as their first tDMARD showed lower rates of type 2 diabetes mellitus (T2DM)-related complications compared to those on other tDMARDs. Reduction in complications: o TNFi-experienced patients: 21 vs. 24 (*p* = 0.046). o tDMARD-naïve patients: A trend toward lower rates (23 vs. 26, *p* = 0.821). o Compared to other non-TNFi biologics: Significant reduction (21 vs. 26, *p* ≤ 0.0001).
**Ruscitti P.** **2019/2021** **Italy** [36]	Multicenter, open-label RCT, (2013 to 2016). 39 RA. anakinra (n = 22), TNFi (n = 17).		***Inclusion criteria***: RA aged >18 years. HbA1c% > 7% and <10%. **Exclusion criteria**: *	**Main outcomes: Change in % of HbA1c and** of RA disease activity. Reduction of antidiabetic drugs. **Follow-up duration**: Until the occurrence of one of the above events	Significant effect of anakinra treatment on the reduction of HbA1c%. TNFi treatment did not show significant effects on the levels of HbA1c% in the same period.
**Chen S.K.** **2020** **US** [37]	Retrospective cohort study (2005–2016). 10 019 RA + DM. ABA (n = 1785). TCZ (n = 759). TNFi (n = 5953). RTX (n = 888). Tofa (n = 634).	IBM MarketScan	***Inclusion criteria***: RA + DM requiring a new dispensing for ABA, a TNFi (ADA, CTZ, ETN, GLM, IFX), RTX, TCZ, Tofa). The ID: date of first study drug dispensing. 1 year of continuous enrollment before the ID. ***Exclusion criteria***: Patients with gestational DM at baseline.	**Main outcome**: DM treatment intensification. **Follow-up duration**: Until the occurrence of one of the above events.	Baseline insulin use: Highest in RTX users (44%), lowest in Tofa users (35%). Comparative risk (vs. ABA): Similar risk for TNFis, RTX, TCZ. Lower risk for Tofa. Non-insulin DM treatment switching: No significant differences between ABA and other bDMARDs
**Song S.-N.** **2013** **Japan** [38]	Prospective cohort study (from June 2008). 93 RA. TCZ (n = 46). TNFi (n = 47). Healthy controls (n = 16).		***Inclusion criteria***: RA ≥ Six months eligible to start bDMARDs. ***Exclusion criteria***: Patients who had received ESAs or iron during the two months before the initiation of the study.	**Main outcomes**: Hb level, iron-related parameters including serum hepcidin, and disease activity. **Follow-up duration**: Monitored before and at 2, 4, 8, and 16 weeks after the initiation of treatment.	TCZ was more effective than TNFis in improving anemia, with a significantly greater increase in Hb levels.
**Abu-Zaid M.H.** **2018** **Egypt** [39]	RCT. 90 RA. MTX+ Placebo, n = 30. MTX + ETA, n = 30. MTX+ ADA, n = 30. 30 healthy (n = 30).		***Inclusion criteria***: RA + ACD. Same dose of folic acid, 10 mg/week. Naïve to treatment with bDMARDs. Not receiving any NSAIDs or any intra-articular or systemic corticosteroid injections within 1 month. ***Exclusion criteria***: Patients with other concomitant hematological diseases (thalassemia and sickle-cell anemia), those with heart, lung, kidney, and liver diseases.	**Main outcomes**: ACD and serum hepcidin levels. **Follow-up duration**: 4 months	TNFi therapies outperformed MTX in reducing disease activity and improving ACD, with a notable drop in hepcidin-25 levels. However, no significant differences were seen between the two TNFi regimens.
**Paul S.K.** **2017** **US** [40]	Retrospective cohort study (2000–2016). 153,788 patients. TCZ (n = 3732). TOFA (n = 3126). obDMARD (n = 55,694). onbDMARD (n = 91,236).	CEMR	***Inclusion criteria***: Age at diagnosis 18 and ≤80 years. Diagnosis date on or after 1 January 2000 to 30 April 2016. No missing data on age, sex, ethnicity. Complete data on Hb and Hct at ID. Initiated a DMARD at the time of diagnosis or during the follow-up period. **Exclusion criteria**: Not mentioned	**Main outcomes**: Change in Hb and Hct. **Follow-up duration**: 2 years.	TCZ demonstrated superior hematologic improvement, particularly in anemic patients, with significantly higher Hb and Hct increases compared to other treatments. Other groups showed minimal to no meaningful changes.
**Silva-Fernandez L.** **2016** **UK** [41]	Retrospective cohort study (2001 to 2012). 425 RA with a prior malignancy. TNFi cohort (n = 243). RTX cohort (n = 23). csDAMRDs (n = 159).	BSRBR-RA	***Inclusion criteria***: RA patients who were commencing a TNFi or RTX as their first biologic and with prior malignancy. ***Exclusion criteria***: Not mentioned	**Main outcomes**: Incidence of cancer in patients with RA and a prior malignancy (new primaries, local recurrence, and metastases were all included as incident cancers). Carcinoma-in situ and non-melanoma skin cancer were excluded, as were benign cancers. **Follow-up duration**: From the ID to 31 May 2013	101 patients developed a new malignancy. TNFis and RTX were associated with lower malignancy rates than csDMARDs, with TNFis showing a statistically significant reduction. RTX had the lowest cancer recurrence rate, suggesting a potential protective effect. csDMARDs had the highest malignancy risk and recurrence rates, warranting caution in high-risk patients.
**Pundole X.** **2020** **USA** [42]	Retrospective cohort study (2002–2014). 431 RA patients with solid malignancies: Age: Not mentioned No bDMARDs (n = 320). bDMARDs (n= 111) (TNFi (ETA, ADA, IFX, CTZ, GLM), non-TNFi (ABA, RTX, TCZ)).		***Inclusion criteria***: RA ≥ 18 years of age, with a histologically confirmed diagnosis of solid malignancy identified from the institutional tumor registry. ***Exclusion criteria***: Nonmelanoma skin cancer with at least 3 months of medical care received.	***Main outcomes***: OS in patients with RA and concomitant solid malignancies. ***Follow-up duration***: From ID until 2016.	Median OS from cancer diagnosis was 16.1 years in patients receiving bDMARDs, most had localized disease, and only 14 (13%) had advanced cancer. No significant differences in OS were observed between patients who received bDMARDs and those who did not.
**Kunishita Y.** **2023** **Japan** [43]	Multicenter, retrospective study. 312 patients with RA were treated with ABT in two hospitals in Yokohama until May 2022. 73 had a history of malignancy.		***Inclusion criteria***: Patients were categorized based on the presence (PM group) or absence (NP group) of previous malignancies. Factors such as age at ABT initiation, methotrexate use, and Steinbrocker stage were considered. ***Exclusion criteria***: Not mentioned	**Main outcomes**: ABT continuation rates and incidence of new malignancies post-ABT initiation were compared between groups, with follow-up until May 2022.	After propensity score matching for age, methotrexate use, and Steinbrocker stage, no significant differences were observed between the NP and PM groups regarding ABT continuation rates and the incidence of new malignancies post-ABT initiation.

**ABA:** Abatacept; **ADA:** Adalimumab; **bDMARDs:** biologic disease-modifying antirheumatic drugs; **BSRBR-RA:** British Society for Rheumatology Biologics Register for Rheumatoid Arthritis; **CEMR**: Centricity Electronic Medical Record; **COPD**: chronic obstructive pulmonary disease; **CTZ**: Certolizumab Pegol; **ED**: emergency department; **ESAs**: erythropoiesis-stimulating agents; **ETA**: Etanercept; **GLM**: Golimumab; **HCRU**: healthcare resource utilization; **ID**: index date (the start of the biologic treatment). **ILD**: interstitial lung disease; **IFX**: Inflixima; **OS**: overall survival; **HbA1c%**: percentage of glycated hemoglobin; **RA**: rheumatoid arthritis; **TCZ**: tocilizumab; **tDMARDs**: targeting disease-modifying antirheumatic drugs. **TNFi**: TNF inhibitor; **Tofa**: Tofacitinib; **T2DM**: Type 2 diabetes mellitus; **UK**: United Kingdom; **US**: United States. *: T2D diagnosed more than 10 years prior to the study; ongoing acute or chronic infection; increased (>30 mg/L) levels of C-reactive protein (CRP); fever; ongoing antibiotic therapy; chronic granulomatous diseases, such as tuberculosis; history of recurrent infections; fasting C-peptide values < 0.5 ng/mL (0.1665 nmol/L); presence of neutropenia (white blood count < 2000/mm^3^) or anemia (hemoglobin < 11 g/dL for men and 10 g/dL for women); the presence of one or more contraindications reported in the data sheet of anakinra or TNFi; the presence of one or more contraindications to MTX; previous ischemic attack or myocardial infarction; heart failure of New York Heart Association (NYHA) class III or IV; hepatic or progressive liver disease (values of alanine aminotransferase/aspartate aminotransferase [ALAT/ASAT] elevated by at least 2-fold compared with normal values); pregnancy or women not using contraceptive measures; breastfeeding; participation in another clinical study up to 6 months before randomization; depressive syndrome or other severe psychiatric illness; the presence of known malignancy; clinically significant history of alcohol abuse or drug addiction; any condition that, in the opinion of the investigator, could preclude the possibility of use of study drugs in compliance with data sheet indications; and any other condition or laboratory parameter that, in the opinion of the investigator, could preclude the participation of the subject in the study.

## Data Availability

No new data were generated.

## List of Abbreviations

ABA	Abatacept
ANA	Anakinra
ACR/EULAR	American College of Rheumatology/European League Against Rheumatism
ADA	Adalimumab
bDMARDS	Biologic Disease-Modifying Antirheumatic Drugs
csDMARDs	Conventional Synthetic Disease-Modifying Antirheumatic Drugs
JAKi	Janus Kinase Inhibitor
CDA	Chronic Disease Anemia
COPD	Chronic Obstructive Pulmonary Disease
ETA	Etanercept
GLM	Golimumab
Hb	Hemoglobin
IFX	Infliximab
ILD	Interstitial Lung Disease
IL6Ri	Interleukin 6 Receptor Inhibitor
JBI	Joanna Briggs Institute
MTX	Methotrexate
PICOS	Population, Intervention, Comparison, Outcomes, and Study Design
PRISMA	Preferred Reporting Items for Systematic Reviews and Meta-Analyses
RA	Rheumatoid Arthritis
RCT	Randomized Controlled Trial
RTX	Rituximab
TCZ	Tocilizumab
tDMARDs	Targeted Disease-Modifying Antirheumatic Drugs
TNFi	Tumor Necrosis Factor Inhibitor
T2DM	Type 2 Diabetes Mellitus
UK	United Kingdom
